# Predictors of change in objectively measured and self-reported health behaviours among individuals with recently diagnosed type 2 diabetes: longitudinal results from the *ADDITION-Plus* trial cohort

**DOI:** 10.1186/1479-5868-10-118

**Published:** 2013-10-23

**Authors:** Laura Kuznetsov, Rebecca K Simmons, Stephen Sutton, Ann-Louise Kinmonth, Simon J Griffin, Wendy Hardeman

**Affiliations:** 1 MRC Epidemiology Unit, University of Cambridge, Box 285, Addenbrooke's Hospital, Hills Road, Cambridge, CB2 0QQ, UK; 2 The Primary Care Unit, Cambridge Institute of Public Health, University of Cambridge, Robinson Way, Cambridge CB2 0SR, UK

**Keywords:** Health behaviour, Behaviour change, Predictors, Type 2 diabetes, Newly diagnosed

## Abstract

**Background:**

There is limited evidence about predictors of health behaviour change in people with type 2 diabetes. The aim of this study was to assess change in health behaviours over one year and to identify predictors of behaviour change among adults with screen-detected and recently clinically diagnosed diabetes.

**Methods:**

*ADDITION-Plus* was a randomised controlled trial of a behaviour change intervention among 478 patients (40–69 years). Physical activity and diet were measured objectively (physical activity at 1 year) and by self-report at baseline and one year. Associations between baseline predictors and behaviour change were quantified using multivariable linear regression.

**Results:**

Participants increased their plasma vitamin C and fruit intake, reduced energy and fat intake from baseline to follow-up. Younger age, male sex, a smaller waist circumference, and a lower systolic blood pressure at baseline were associated with higher levels of objectively measured physical activity at one year. Greater increases in plasma vitamin C were observed in women (beta-coefficient [95% CI]: beta = −5.52 [−9.81, -1.22]) and in those with screen-detected diabetes (beta = 6.09 [1.74, 10.43]). Younger age predicted a greater reduction in fat (beta = −0.43 [−0.72, -0.13]) and energy intake (beta = −6.62 [−13.2, -0.05]). Patients with screen-detected diabetes (beta = 74.2 [27.92, 120.41]) reported a greater increase in fruit intake. There were no significant predictors of change in self-reported physical activity. Beliefs about behaviour change and diabetes did not predict behaviour change.

**Conclusions:**

Older patients, men and those with a longer duration of diabetes may need more intensive support for dietary change. We recommend that future studies use objective measurement of health behaviours and that researchers add predictors beyond the individual level. Our results support a focus on establishing healthy lifestyle changes early in the diabetes disease trajectory.

## Background

Regular physical activity (PA) and healthy eating confer metabolic and cardiovascular benefit for people with type 2 diabetes [1-4]. Exercise intervention significantly increases insulin response, improves glycaemic control, and decreases plasma triglycerides in people with established diabetes, while weight reduction significantly improves glycaemic control and lowers blood pressure and CVD risk [1]. Randomised controlled trials (RCTs) evaluating PA/or diet interventions among individuals with recently diagnosed diabetes (e.g. diagnosed in the last three years) report an improvement in glycaemic control and modelled CVD risk [5-7].

Despite the established benefits of a healthy lifestyle and its importance for the clinical management of diabetes, many people who have been diagnosed find it difficult to achieve and maintain changes in health behaviour [8,9]. There may be a window of opportunity to facilitate health behaviour change soon after diagnosis. This may vary according to the time elapsed since diagnosis and whether patients are detected through screening or diagnosed in routine clinical practice [10]. Recognising patients who are more or less likely to change their health-related behaviour after being diagnosed with diabetes, may offer the possibility of tailored support and inform the development of behavioural interventions. However, few previous studies have examined predictors of change in PA and diet among people with recently diagnosed diabetes. For example, results from an RCT examining a brief self-management intervention to support patients with recently diagnosed diabetes (n = 180) to achieve sustained improvements in their exercise and diet showed that participants with a higher proactive competence (e.g. initiating health behaviours, dealing with potential barriers to goal-maintenance) at baseline reported greater self-management and improvement in exercise and diet at 12 months [11]. Baseline exercise behaviour, body mass index (BMI) and dietary behaviour were also positively associated with exercise and diet at follow-up. A study with 6 months follow-up in a cohort of newly diagnosed patients (n = 204) revealed that younger individuals, men, and those who had positive beliefs about the long-term consequences of exercise behaviour were more likely to increase their PA [12].

The interpretation of previous studies is limited by the measurement of health behaviours using self-report [11-13], small sample size [11-13], short-term follow-up [12,13], and examination of a limited range of predictors [11-13]. This makes it difficult to generalise results to patients with recently diagnosed diabetes. Using data from the *ADDITION-Plus* trial, which evaluated a behaviour change intervention among recently diagnosed diabetes patients with objective measurement of health behaviours and one year follow-up, we aimed to (i) assess change in health behaviours over one year and (ii) examine associations between baseline socio-demographic, clinical and psychological predictors, and change in health behaviours.

## Methods

The design and rationale for the *ADDITION-Plus* trial have been reported previously [14]. In brief, *ADDITION-Plus* (2002–2007) is a randomised controlled trial nested within the intensive treatment arm of the *ADDITION-Cambridge* study, which evaluated the efficacy of a facilitator-led, theory-based behaviour change intervention over and above intensive general practice team-led treatment among recently diagnosed patients with diabetes. Thirty four general practices (GP) in the East of England participated. Eligible individuals were those aged 40–69 years diagnosed with diabetes following screening in the *ADDITION* study [15] or clinically diagnosed during the three previous years in participating GP surgeries. Exclusion criteria included pregnant or lactating women or those with a likely prognosis of less than one year. Eligible participants (n = 478) were individually randomised to receive either intensive treatment alone (n = 239), or intensive treatment plus a behaviour change intervention delivered by trained facilitators at the patient’s practice (n = 239). The intervention was designed to build on the diabetes education delivered by practice nurses and included a one-hour introductory meeting followed by individually tailored six 30-minute meetings and four brief phone calls during the one year period. Baseline measurements were carried out on all eligible patients including the completion of standardised self-report questionnaires, physiological and anthropometric measures, and venesection (including assessment of plasma vitamin C). Full details of these measurements are published elsewhere [14]. Similar measurements were conducted one year after recruitment, as well as objectively measured PA using a combined heart rate and accelerometry monitor. All participants gave written informed consent, and the study was approved by the Eastern Multi-Centre Research Ethics Committee (reference number: 02/5/54). The trial is registered as ISRCTN 99175498.

### Socio-demographics

Standardised self-report questionnaires were used to collect information on age, sex, age left full-time education, employment and marital status, social class and ethnicity.

### Clinical measures

Clinical measures were collected by trained staff following standard operating procedures. Waist circumference was estimated as the average of two measurements taken halfway between the lowest point of the rib cage and the anterior superior iliac crests while standing. Blood pressure was calculated as the mean of three measurements performed after 10 minutes rest, while participants were seated with the cuff on the predominant arm at the level of the heart, using an automatic sphygmomanometer (Omron M4, UK). Glycosylated haemoglobin (HbA_1c_) was analysed in venous samples by ion-exchange high-performance liquid chromatography (Tosoh Bioscience, Redditch, UK). Serum total cholesterol was measured using enzymatic techniques (Dade Behring Dimension analyser, Newark, USA). Recommendations for the treatment and management of diabetes include a reduction and/or maintenance in levels of blood glucose (HbA_1c_ between 6.5 and 7.5%), blood lipids (total-cholesterol <4.0 mmol/L) and blood pressure (systolic <140 mmHg) [16].

### Health behaviour measures

Objectively measured PA was assessed at one year using a combined heart rate and accelerometry monitor (Actiheart, CamNtech, Cambridge, UK), which was worn continuously for at least four days [17]. A graded treadmill walk test was used to individually calibrate heart rate. Heart rate data collected during the free-living period were processed [18] and activity intensity (J/min/kg) was estimated using a branched equation framework [19]. Resulting time-series data were summarised into PA energy expenditure (PAEE, in kJ/kg/d), whilst minimising diurnal information bias caused by non-wear periods (segments of non-physiological data). Data from participants who did not complete individual calibration were processed using an age, sex, beta-blocker, and sleeping heart rate adjusted group calibration equation for the translation of heart-rate into activity intensity [19]. Quantification of plasma vitamin C, a robust measure of fruit and vegetable intake, was assessed at baseline and one-year using a Fluoroskan Ascent FL fluorometer [20,21].

Self-reported PA was assessed at baseline and one year using the validated EPAQ2 questionnaire [22] and expressed as total metabolic equivalent task (MET) hours per day. Dietary intake was assessed at baseline and one year using a validated food-frequency questionnaire (FFQ) [21]. The FFQ questionnaire includes questions about usual consumption of 130 foods, assessed with a 9-point scale ranging from “never or less than once/month” to “6 times per day”; and additional free text questions to categorise breakfast cereals, total fat and fatty acid consumption. The data are then converted into an amount for each nutrient profile and average daily nutrient intake, by reference to a regularly updated reference source for the nutritional content of food products. We used self-reported fat intake (grams/day), energy intake (kcal/day), fruit intake (grams/day) and vegetable intake (grams/day).

### Psychological measures

Anxiety was measured using the six item Spielberger short form State Anxiety Inventory (STAI) [23], with higher scores indicating higher state anxiety (baseline Cronbach’s α = 0.79). Self-rated general health was measured using a single item on a 5-point Likert-type scale ranging from ‘excellent’ to ‘poor’, with a higher score indicating worse health.

A questionnaire based on the Theory of Planned Behaviour (TPB) [24] assessed intention to become more active and to eat a lower fat diet (e.g. ‘I intend to be more physically active/eat a lower fat diet in the next 12 months’), perceived control (e.g. ‘I am confident that I could be more physically active/eat a lower fat diet in the next 12 months, if I wanted to’) and beliefs about these behaviours (e.g. ‘If I was more physically active/eat a lower fat diet in the next 12 months, it is likely that that my health would improve’). Two items were used for each construct measured on a 5-point Likert-type scales ranging from ‘strongly disagree’ to ‘strongly agree’, with higher scores indicating more positive beliefs. Baseline Cronbach’s alpha indicated satisfactory reliability of the scales (range from 0.77 to 0.89). To assess beliefs about diabetes consequences (e.g. ‘My diabetes is a serious condition’, Cronbach’s α = 0.74) and treatment control (e.g. ‘My treatment can control my diabetes’, Cronbach’s α = 0.56) subscales (11 items) of the Illness Perception Questionnaire-Revised (IPQ-R) were used [25]. The items were measured on a 5-point Likert-type scale ranging from ‘strongly disagree’ to ‘strongly agree’, with higher scores indicating stronger perceptions of diabetes consequences and treatment effectiveness.

### Data analyses

Trial analyses showed that the behaviour change intervention did not lead to change in health behaviours or cardiovascular risk factors over and above intensive treatment alone [26]. Consequently, the two trial arms were pooled and a cohort analysis conducted. Descriptive characteristics were summarised using means (±SDs) or frequencies. We compared change in health behaviours between baseline and follow-up separately for men and women using a paired t-test for continuous data. Multivariable linear regression analysis was conducted to examine associations between the baseline predictors and health behaviours at one year, adjusting for baseline health behaviour (except for PAEE), trial group and all predictor variables. Tolerance statistics and variance inflation factors were used to test for multicollinearity and were within acceptable limits for all variables. The residuals of all regression models were examined to ensure that they were approximately normally distributed. We analysed change by including values of outcomes at one year in the regression model, adjusting for baseline values [27]. Sensitivity analyses showed that using a change score whilst adjusting for baseline values produced identical beta coefficients and p-values, except for the baseline covariate. All regression results are presented as unstandardised β-coefficients. Statistical analyses were performed using Stata/SE 12.0 (Stata-Corp, College Station, TX) and SPSS for Windows 19.0 (SPSS, Inc., Chicago, IL). Statistical significance was set at *P* < 0.05.

## Results

The mean age of participants was 60 years, 62% were male and the cohort was largely Caucasian (Table 1). The cohort exhibited an adverse cardiovascular risk profile with large waist circumferences and elevated systolic blood pressure, HbA_1c_ and total cholesterol values. Half of the participants had screen-detected diabetes and half were clinically diagnosed in the previous three years. Participants reported relatively strong intentions, perceived behavioural control, and behavioural beliefs towards changing behaviour, high diabetes treatment control perception, good general health, and relatively low state anxiety. There were no significant baseline differences between those attending the one-year health assessment and those who did not (n = 23) for age, sex, social class, waist circumference, total cholesterol, and for the majority of psychological predictors. However, those who did not attend follow-up had a higher baseline HbA_1c_ (mean 7.8 ± 1.88%) compared to those who did attend (7.1 ± 1.44%, *p* = 0.032).

**Table 1:** **Baseline socio-demographic, clinical and psychological characteristics of the *ADDITION-Plus *** **cohort**^**a**^

**Variables**	**Mean ± SD**
**Socio-demographic characteristics**	
Age (years), n (mean ± SD)	478 (59.7 ± 7.5)
Male gender, n (%)	298 (62.3)
Full-time education finished at >16 years, n (%)	291 (61.7)
In full- or part-time employment, n (%)	245 (51.4)
Married, n (%)	359 (75.4)
Social class, n (%)	
Managerial/professional occupations	184 (39.0)
Intermediate occupations	120 (25.4)
Routine/manual occupations	168 (35.6)
Caucasian ethnicity, n (%)	466 (97.5)
**Clinical characteristics**	
Waist circumference (cm), n	477 (110.8 ± 13.8)
Systolic blood pressure (mmHg), n	478 (136.1 ± 19.23)
Diastolic blood pressure (mmHg), n	478 (80.3 ± 10.42)
HbA_1c_ (%), n	472 (7.1 ± 1.47)
Total cholesterol (mmol/l), n	474 (4.9 ± 1.08)
Screen-detected type 2 diabetes, n (%)	239 (50.0)
**Psychological characteristics (1 to 5)**	
*Intention*	
Physical activity, n	463 (3.7 ± 0.78)
Lower fat diet, n	464 (3.7 ± 0.78)
*Perceived behavioural control*	
Physical activity, n	463 (3.8 ± 0.86)
Lower fat diet, n	465 (3.7 ± 0.87)
*Behavioural beliefs*	
Physical activity, n	467 (4 ± 0.68)
Lower fat diet, n	464 (3.8 ± 0.73)
*Illness perception (1 to 5)*	
Diabetes treatment control, n	451 (3.8 ± 0.49)
Diabetes consequences, n	454 (2.9 ± 0.65)
*State anxiety (20 to 80),* n	467 (32.5 ± 11.29)
*Self-rated health (1 to 5)*, n	468 (3.2 ± 0.84)

Values are means ± SD unless otherwise indicated.

^a^Sample sizes differ due to missing data.

### Health behaviours at one-year follow-up and change over the year

Men had higher levels of PAEE than women at one year. Only women reported significant increases in self-reported PA from baseline to one year (0.85 metabolic equivalent h/d which is equivalent to 13 min/brisk walking/d). Both sexes reported reduced energy and fat intake, and increased fruit intake. Men also reported increases in vegetable intake. There were corresponding increases in plasma vitamin C levels in both men and women from baseline to follow-up (Table 2).

**Table 2:** **Change in health behaviours between baseline and one year in the *ADDITION-Plus *** **cohort stratified by sex**^**a**^

**Variables**	**Men**			**Women**		
	**Baseline**	**1 year**	**Difference (95% CI)**	**Baseline**	**1 year**	**Difference (95% CI)**
**Objectively measured health behaviours**						
Physical activity energy expenditure (kJ/kg/d), n	Not measured	270, 37.8 ± 18.1	-	Not measured	160, 29 ± 13.6	-
Plasma vitamin C (μmol/l), n	260, 47.1 ± 19.1	260, 49.9 ± 21.9	2.78 (0.21, 5.35)*	152, 55.7 ± 20.8	152, 59.6 ± 20.8	3.91 (0.37, 7.45)*
**Self-reported health behaviours**						
Total physical activity (MET h/d), n	280, 13.2 ± 8.2	280, 13.5 ± 8.4	0.25 (−0.57, 1.07)	168, 10.1 ± 5.5	168, 10.9 ± 5.7	0.85 (0.11, 1.59)*
Fat intake (g/day), n	279, 71.5 ± 30.6	279, 61.5 ± 22.2	−10.04 (−13.06, -7.01)***	166, 63.4 ± 28.2	166, 58.6 ± 23.5	−4.82 (−8.69, -0.95)*
Energy intake (kcal/d), n	279, 2004 ± 657	279, 1790 ± 498	−214 (−279, -149)***	166, 1776 ± 594	166, 1681 ± 504	−95 (−178, -12)*
Fruit intake (g/d), n	261, 253.4 ± 199.7	261, 292.7 ± 207.3	39.26 (12.64, 65.89)**	155, 303 ± 189.4	155, 343 ± 229.3	40.03 (5.85, 74.2)*
Vegetable intake (g/d), n	259, 209.8 ± 114.3	259, 229.2 ± 146	19.41 (5.19, 33.64)**	145, 277.5 ± 180.9	145, 277.6 ± 133.7	0.09 (−26.17, 26.36)

Values are means ± SD.

^a^Sample sizes differ due to missing data.

* *p* < 0.05, ** *p* < 0.01, *** *p* < 0.001.

95% CI, confidence interval.

### Multivariable predictors of health behaviour change and levels

Younger patients, men, those with smaller waist circumference and with lower systolic blood pressure and higher diastolic blood pressure at baseline had higher PAEE levels at one year.

Women and patients with screen-detected diabetes were more likely to exhibit a larger increase in plasma vitamin C. Younger patients and those with clinically diagnosed diabetes were more likely to report larger declines in their fat intake. Younger patients also reported larger decreases in energy intake. Screen-detected patients and those reporting better health at baseline were more likely to report increases in their fruit intake. There were no significant associations between any baseline predictors and change in self-reported PA and vegetable intake. Attitudes, beliefs and anxiety levels did not predict change in health behaviours in multivariable analyses (Table 3).

**Table 3:** **Multivariable associations between baseline characteristics and health behaviours at one year in the *ADDITION-Plus *** **cohort**

**Potential predictors**	**PAEE (kJ/kg/d), n = 378**	**Plasma vitamin C (μmol/l), n = 363**	**Self-reported physical activity (MET h/d), n = 390**	**Self-reported fat intake (g/d), n = 390**	**Self-reported energy intake (kcal/d), n = 390**	**Self-reported fruit intake (g/d), n = 365**	**Self-reported vegetable intake (g/d), n = 358**
**Socio-demographic characteristics**							
Age (years)	−0.72 (−0.98, -0.47)***	0.3 (−0, 0.6)	−0.06 (−0.16, 0.33)	−0.43 (−0.72, -0.13)**	−6.62 (−13.2, -0.05)*	−1.17 (−4.33, 1.98)	−0.52 (−2.54, 1.49)
Sex (women = ref.)	10.9 (7.35, 14.51)***	−5.52 (−9.81, -1.22)*	0.46 (−0.88, 1.81)	0.19 (−4.39, 4.01)	9.94 (−83.91, 103.78)	−37 (−82.36, 8.34)	−4.46 (−34.01, 25.09)
Social class (managerial/professional occ. = ref.)							
Intermediate	0.63 (−3.53, 4.79)	0.36 (−4.69, 5.41)	0.41 (−1.13, 1.95)	0.82 (−4.17, 5.81)	11.4 (−99.3, 122.07)	−16.9 (−70.09, 36.34)	−0.09 (−33.85, 33.66)
Routine/manual	2.26 (−1.47, 6)	−3.24 (−7.7, 1.23)	0.21 (−1.18, 1.59)	−1.36 (−5.75, 3.03)	−72.3 (−169.61, 24.97)	−4.5 (−52.03, 43.02)	−3.06 (−33.0, 26.87)
**Clinical characteristics**							
Waist circumference (cm)	−0.33 (−0.45, -0.2)***	−0.1 (−0.26, 0.05)	−0.03(−0.07, 0.02)	−0.11 (−0.26, 0.04)	−2.97 (−6.28, 0.34)	0.49 (−1.11, 2.08)	−0.32 (−1.33, 0.69)
Systolic blood pressure (mmHg)	−0.18 (−0.31, -0.06)**	−0.05 (−0.2, 0.1)	0.02 (−0.02, 0.07)	−0.09 (−0.24, 0.06)	−1.2 (−4.49, 2.08)	0.9 (−0.68, 2.49)	−0.01 (−1.01, 0.98)
Diastolic blood pressure (mmHg)	0.23 (0, 0.46)*	0.03 (−0.25, 0.31)	−0.01 (−0.1, 0.07)	0.06 (−0.21, 0.34)	0.68 (−5.34, 6.69)	−1.56 (−4.45, 1.32)	−0.24 (−2.06, 1.58)
HbA_1c_ (%)	−0.25 (−1.36, 0.86)	−0.29 (−1.67, 1.09)	0.31 (−0.11, 0.72)	−0.37 (−1.71, 0.98)	−16.0 (−45.67, 13.67)	−0.95 (−15.41, 13.51)	−1.77 (−11.64, 8.1)
Cholesterol (mmol/l)	0.37 (−1.22, 1.96)	−1.61 (−3.54, 0.33)	−0.28 (−0.88, 0.32)	−0.72 (−2.62, 1.18)	−32.8 (−74.95, 9.31)	−18.9 (−39.15, 1.45)	3.91 (−9.19, 17.02)
Diabetes diagnosis (clinically diagnosed = ref.)	2.91 (−0.59, 6.42)	6.09 (1.74, 10.43)**	1.04 (−0.27, 2.35)	−4.9 (−9.21, -0.6)*	−56.5 (−151.76, 38.81)	74.2 (27.92, 120.41)**	10.2 (−19.43, 39.82)
**Psychological characteristics**							
*Intention*							
Physical activity	−0.37 (−3.41, 2.68)	-	0.69 (−0.46, 1.84)	-	-	-	-
Lower fat diet	-	−1.89 (−5.68, 1.89)	-	−1.01 (−4.81, 2.78)	−26.2 (−110.29, 57.83)	−20.8 (−62.08, 20.44)	22.5 (−3.14, 48.04)
*Perceived behavioural control*							
Physical activity	1.04 (−1.47, 3.55)	-	0.04 (−0.9, 0.98)	-	-	-	-
Lower fat diet	-	0.22 (−2.86, 3.3)	-	−0.98 (−4.04, 2.09)	−7.52 (−75.32, 60.27)	17.7 (−16.12, 51.41)	−5.49 (−26.22, 15.24)
*Behavioural beliefs*							
Physical activity	−1.06 (−4.13, 2.02)	-	−0.7 (−1.84, 0.43)	-	-	-	-
Lower fat diet	-	0.06 (−3.67, 3.79)	-	0.62 (−3.11, 4.35)	19.6 (−62.88, 102.09)	−5.21 (−45.29, 34.87)	−8.66 (−34.28, 16.95)
*Illness perception (1 to 5)*							
Diabetes treatment control	2.33 (−1.25, 5.91)	3.7 (−0.64, 8.04)	−0.38 (−1.71, 0.94)	0.24 (−3.99, 4.48)	9.49 (−84.44, 103.43)	−3.08 (−48.07, 41.92)	3.54 (−24.58, 31.67)
Diabetes consequences	1.51 (−1, 4.03)	1.43 (−1.65, 4.51)	0.58 (−0.38, 1.53)	−1.8 (−4.89, 1.29)	6.62 (−61.81, 75.04)	21.8 (−11.66, 55.18)	13.1 (−7.54, 33.73)
*State anxiety (20 to 80)*	−0.07 (−0.22, 0.08)	−0.12 (−0.3, 0.06)	−0.04 (−0.09, 0.02)	−0.06 (−0.25, 0.12)	−1.2 (−5.26, 2.86)	−0.81 (−2.74, 1.13)	0.15 (−1.07, 1.38)
*Self-rated health (1 to 5)*	−0.58 (−2.73, 1.58)	−0.6 (−3.19, 1.98)	−0.19 (−1.0, 0.62)	0.46 (−2.08, 3)	−2.66 (−59.09, 53.76)	−28 (−55.33, -0.69)*	−10.3 (−27.9, 7.36)

Values are unstandardised b-coefficients (95% confidence interval).

All models were simultaneously adjusted for included predictors as well as for baseline behaviour (except for PAEE) and trial arm.

PAEE, physical activity energy expenditure.

-, not included in the model.

* *p* < 0.05, ** *p* < 0.01, *** *p* < 0.00.1.

## Discussion

Younger age and diabetes detected through screening emerged as the most consistent predictors of dietary change over the year of study, and objective physical activity at one year. Our finding that younger patients engaged in higher levels of physical activity supports previous literature [28]. Patients with screen-detected diabetes were diagnosed more recently than clinically diagnosed patients. The diagnosis may have acted as a cue to adopt healthier behaviours over the year of our study, whereas patients with clinically diagnosed diabetes may have faced challenges to maintain any changes in behaviour made soon after diagnosis. Our findings contribute to previous literature by suggesting the existence of a window of opportunity to facilitate behaviour change early in the disease trajectory.

There were significant improvements in self-reported PA in women, and in self-reported and objectively measured dietary variables in men and women over one year in this cohort of recently diagnosed diabetes patients. Intervention studies focusing on lifestyle change among people with recently diagnosed [7,11] and established diabetes have also reported positive changes in self-reported PA and diet over 12 months [29].

There were significant associations between younger age, male sex, a smaller waist circumference, and lower systolic blood pressure values at baseline and higher PAEE levels at one year. These results are partially in agreement with other studies. A study among offspring of people with diabetes (mean age 40 years) identified male sex and a higher level of baseline fitness as predictors of change in objectively measured PA [30]. A review of correlates of adults’ participation in self-reported PA showed that PA participation was consistently higher among men than women and was inversely associated with age [28]. A cohort study revealed that adults (≥55 years) with a large waist circumference were more likely to be physically inactive than those with smaller waists [31]. An association was also observed between baseline diastolic blood pressure and PAEE at one year, but this was of borderline statistical significance.

We did not find any significant baseline predictors of change in self-reported PA. Previous studies identified the following predictors of self-reported PA in patients with recently diagnosed diabetes: age [12], sex [12], BMI [11,13], future-oriented thinking [12], proactive competence [11], and baseline exercise behaviour [11]. These findings are similar to our results regarding objectively measured PA levels at one year, but not with change in self-reported PA. Differences may be due to length of follow-up (short-term predictors of change may differ from long-term predictors), the larger age ranges targeted by other interventions, and the difference in measurement method [12,13].

Greater increases in plasma vitamin C levels were observed in women, which is consistent with other studies that have shown higher plasma vitamin C concentrations in women [32,33]. We observed a greater change in plasma vitamin C levels and in fruit intake among patients with screen-detected diabetes than in clinically diagnosed patients. This might reflect a greater capacity for change in dietary behaviour in the first year after diagnosis among screen-detected patients, while clinically diagnosed patients have lived with the condition for longer and may have already made significant changes to their diet. While the difference in the increase in plasma vitamin C levels between screen- and clinically-diagnosed patients was small (6 μmol/l), the difference between the 25th and 75th percentiles was 24 μmol/l, suggesting that patients in the top quartile of plasma vitamin C consumed one more portion of fruit and vegetables per day compared with those in the bottom quartile. A similar difference was seen in self-reported fruit intake, where screen-detected diabetes patients increased on average 74 g/d (which equals a fresh apricot) compared with clinically diagnosed diabetes patients.

*ADDITION-Plus* participants who reported better health at baseline were also more likely to report increases in fruit intake. A cross-sectional study among US adults indicated that the odds of consuming ≥5 servings of fruits and vegetables per day were higher among those who rated their health as excellent/very good compared to those who rated their health as poor [34]. We did not observe any significant associations between baseline predictors and change in self-reported vegetable intake in multivariable analysis.

Younger patients were more likely to report a greater reduction in fat and energy intake. Other studies among patients with recently diagnosed diabetes showed that only baseline dietary behaviour [11,12] and proactive competence [11] predicted fat consumption at follow-up. Having a clinical diagnosis of diabetes was a predictor of greater decrease in fat intake (approx. 5 g/d which equals, for example, one teaspoon of mayonnaise) indicating a better management of fat intake among patients who lived with the condition for longer.

Intention, perceived behavioural control and beliefs about becoming more physically active and eating a low fat diet did not predict change in objectively measured or self-reported behaviours in the *ADDITION-Plus* cohort. These findings replicate results in a study by Thoolen et al. [11] in which exercise and diet intentions did not predict these behaviours at follow-up in patients with recently diagnosed diabetes. Lack of associations is unlikely to be due to lack of power as our sample size was large compared to most studies in this area. Instead it may be due to inaccurate or unrealistic beliefs at baseline when patients had limited experience with behaviour change, the one-year interval between measurement of beliefs and behaviour, or behaviour change through automatic routes (e.g. activation of previous goals) rather than reflective routes [35]. Although previous research suggested that the TPB is useful for the prediction of health behaviours in the general population [36], studies of prediction of behaviour change using the TPB are few, and findings for people at risk for diabetes are inconsistent [37,38]. Our results suggest that the TPB might not be a useful framework for predicting changes in health-related behaviours in individuals with recently diagnosed diabetes. Other psychological predictors also did not predict change in health-related behaviours in our cohort. This might be partially attributed to the low reliability of some measures (Cronbach’s alpha at baseline was 0.56 for treatment control) or common method variance [39]. When a behaviour and its determinants are measured using the same method e.g. by self-report questionnaire, associations may be due, at least in part, to commonality in response patterns to these measures. When the behaviour is measured by a different method, e.g. objectively, part of the correlation explained by common method variance disappears, leading to lower or non-significant associations. Some studies have shown, for example, that psychological determinants of dietary behaviour predict self-rated fruit and vegetable intake (asking people to rate their own fruit and vegetable consumption) better than intake assessed by FFQ (general food intake questionnaire) [40,41]. Finally, since it has been shown that environmental factors are significantly related to health behaviours [28], and that social support might be important in health behaviour change [28,42], it would be desirable to incorporate these variables as correlates of behaviour change in future studies.

The present study has several strengths. *ADDITION-Plus* included objective and self-report measurement of two key health behaviours over 12 months in a well-defined group of patients at high cardiovascular risk who could benefit from positive changes in diet and PA. The use of an objective measure of PA, which has been extensively validated in the laboratory and during free-living conditions [43,44], reduces the error and bias commonly associated with self-report measures. There was a high follow-up rate (95%) and a wide range of potential predictors from a variety of domains were examined. The study also has several limitations. While the sample was population-based, it was largely Caucasian and middle-aged, which restricts generalizability to other populations. Other limitations include the fact that PAEE was only measured at one year, and that self-reported health behaviours may be subject to recall and social desirability bias. Furthermore, we also explored a number of associations and conducted multiple significance tests, which mean that our results should be interpreted with caution as some significant associations may have occurred by chance (alpha inflation).

## Conclusions

We observed significant improvements in self-reported PA in women, and in self-reported and objectively measured dietary variables in men and women over one year in this cohort of recently diagnosed diabetes patients. Younger age, female sex and a screen-detected diagnosis of diabetes were strong predictors of improved dietary behaviours, suggesting that older patients, men and those with a longer duration of diabetes may need more support for dietary change. We did not identify any baseline predictors of self-reported physical activity change but did observe associations with objectively measured PA at one year. We recommend that future studies use objective measurement of health behaviours and that researchers add predictors beyond the individual level. Our results support a focus on establishing healthy lifestyle changes early in the diabetes disease trajectory.

## Competing interests

No potential conflicts of interest relevant to this article were reported by the authors of this paper.

## Authors’ contributions

WH, SS and RKS conceived the study question. LK analysed and interpreted the data, and produced the first draft of the manuscript. All authors contributed to the drafting of the manuscript, critically revised the manuscript for important intellectual content and approved the final version.
